# *Plasmodium* infection and its association with biochemical and haematological parameters in free-living *Alouatta guariba clamitans* (Cabrera, 1940) (Primates: Atelidae) in Southern Brazil

**DOI:** 10.1590/0074-02760190210

**Published:** 2020-01-31

**Authors:** Ana Júlia Dutra Nunes, Denise Anete Madureira de Alvarenga, Julio Cesar de Souza, Amanda Rezende Peruchi, Gustavo Henrique Pereira Gonçalves, Zelinda Maria Braga Hirano, Cristiana Ferreira Alves de Brito, Marta Jussara Cremer

**Affiliations:** 1 Universidade da Região de Joinville, Programa de Saúde e Meio Ambiente, Joinville, SC, Brasil; 2 Fundação Oswaldo Cruz-Fiocruz, Instituto René Rachou, Belo Horizonte, MG, Brasil; 3 Universidade Regional de Blumenau, Centro de Pesquisas Biológicas de Indaial, Projeto Bugio, Indaial, SC, Brasil; 4 Universidade da Região de Joinville, Laboratório de Ecologia e Conservação de Tetrápodes Marinhos e Costeiros, São Francisco do Sul, SC, Brasil

**Keywords:** Plasmodium simium, Plasmodium brasilianum, Alouatta guariba clamitans, endangered species, malaria, primate diseases

## Abstract

**BACKGROUND:**

The influence of *Plasmodium* spp. infection in the health of Southern brown howler monkey, *Alouatta guariba clamitans*, the main reservoir of malaria in the Atlantic Forest, is still unknown.

**OBJECTIVES:**

The aim of this study was to investigate the positivity rate of *Plasmodium* infection in free-living howler monkeys in an Atlantic Forest fragment in Joinville/SC and to associate the infection with clinical, morphometrical, haematological and biochemical alterations.

**METHODS:**

Molecular diagnosis of *Plasmodium* infection in the captured monkeys was performed by *Nested*-polymerase chain reaction (PCR) (18S rRNA and *coxI*). Haematological and biochemical parameters were compared among infected and uninfected monkeys; clinical and morphometrical parameters were also compared.

**FINDINGS:**

The positivity rate of *Plasmodium* infection was 70% among forty captured animals, the highest reported for neotropical primates. None statistical differences were detected in the clinical parameters, and morphometric measures comparing infected and uninfected groups. The main significant alteration was the higher alanine aminotransferase (ALT) levels in infected compared to uninfected monkeys.

**MAIN CONCLUSIONS:**

Therefore, *Plasmodium* infection in howler monkeys may causes haematological/biochemical alterations which might suggest hepatic impairment. Moreover, infection must be monitored for the eco-epidemiological surveillance of malaria in the Atlantic Forest and during primate conservation program that involves the animal movement, such as translocations.

Malaria is an important parasitic disease caused by obligate intracellular protozoa of the genus *Plasmodium* (Coccidia, Haemosporida: Plasmodiidae), which infects mammals, birds, and reptiles. The species that cause infection in humans are *Plasmodium falciparum* (Welch, 1897), *Plasmodium vivax* (Grassi & Feletti, 1890), *Plasmodium malariae* (Laveran, 1881), *Plasmodium ovale* (Stephens, 1922), and *Plasmodium knowlesi* (Sinton and Mulliga, 1933), which occurs in Southeast Asia and, originally, infects non-human primates in the Old World.^1^

Infection by *Plasmodium* in non-human primates occurs in several prosimians, apes, Old World and New World primate species.^2^ Twenty-eight species of *Plasmodium* have been described infecting non-human primates, but only two parasite New World primates (NWPs) in different countries of the Central and South America: *Plasmodium brasilianum* (Gonder & Berenberg-Gossler, 1908), and *Plasmodium simium* (Fonseca, 1951). These parasites are morphologically, genetically and immunologically similar to the human parasites *P. malariae*, and *P. vivax*, respectively.^3^ ^,^ ^4^ Lalremruata et al. and Brasil et al. molecularly characterised *P. brasilianum* and *P. simium* infections in humans and NWPs, suggesting their zoonotic transmission.^5^ ^,^ ^6^

In 2017, around 219 million human malaria cases were recorded worldwide, causing about 435,000 deaths, of which around 90% of the cases and deaths occurred in African continent.^1^ In Brazil, 217,928 cases of malaria were registered in 2017 (Available in: https://public.tableau.com/profile/mal.ria.brasil#!/). In the extra-Amazon Region, 310 confirmed cases of *P. falciparum* or mixed malaria, and 422 confirmed cases of *P. vivax* were reported in 2018. Brasil et al. reported a *P. simium* outbreak in the mountain region of the Rio de Janeiro State, suggesting a zoonotic transmission of malaria in the Atlantic Forest region.^6^ Zoonotic malaria represents a unique problem for the efforts in malaria control and hinders eventual elimination of malaria, because non-human primates may act as reservoirs for malaria in forest regions.^2^ ^,^ ^3^ ^,^ ^6^

A large number of NWP species are susceptible to malaria infection, and amongst the species infected by *P. simium* and *P. brasilianum* the howler monkey, *Alouatta guariba clamitans*, is one of the most important.^3^ This howler monkey is endemic of the Atlantic Forest and is considered vulnerable in Santa Catarina State and also in Brazil. According to the International Union for Conservation of Nature (IUCN) (Available from: https://www.iucn.org), the subspecies is threatened mainly by habitat loss or fragmentation and death caused by diseases, especially Yellow Fever (YF). Although *Plasmodium* infection has already been described in these animals,^3^ ^,^ ^7^ ^,^ ^8^ the impact on animal health and conservation has not been elucidated yet.

One of the few reports of suggestive symptoms of malaria in NWPs was described by Costa et al. for a captive howler monkey in the municipality of Indaial, Santa Catarina, South Brasil.^8^ The primate was infected by *P. simium*, but other infections were not investigated.^8^ The animal showed anorexia, weakness, apathy, intermittent muscle tremors, pale mucous membranes, mild dehydration, and loss of muscle mass and body weight. Additionally, it presented some haematological and biochemical alterations, such as anemia, leucopenia with neutropenia, and severe thrombocytopenia. Biochemical analyses showed a significant increase in urea, alanine aminotransferase (ALT) and aspartate aminotransferase (AST), hypoalbuminemia, and hypoproteinemia.^8^

The present study aimed at determining the positivity and identifying the species of *Plasmodium* responsible for the infection in a population of free-living *A. guariba clamitans* in a fragment of forest in urban perimeter in South Brazil. Furthermore, variations in haematological and biochemical values, as well as morphometric and clinical parameters were evaluated according to the *Plasmodium* spp. infection.

## MATERIALS AND METHODS

*Study area -* The study was performed in a 163 ha fragment of ombrophilous dense lowland forest, located in a private property in the northern region of the city of Joinville, in Santa Catarina State, South Brazil (26º 14’ 41.78” S; 48º 53’ 02.87” W) (Figure). This municipality is the largest in the state, with 583,144 people and 1,126,106 km² of territory (Instituto Brasileiro de Geografia e Estatística - IBGE) (http://www.ibge.gov.br). The fragment is located in the border between an industrial and a rural area, being part of the River Basin of the Rio do Braço. In this area, the density of the brown howler monkey was estimated in 0.82 individuals/hectare, with a total of 133 animals.

**Figure:**
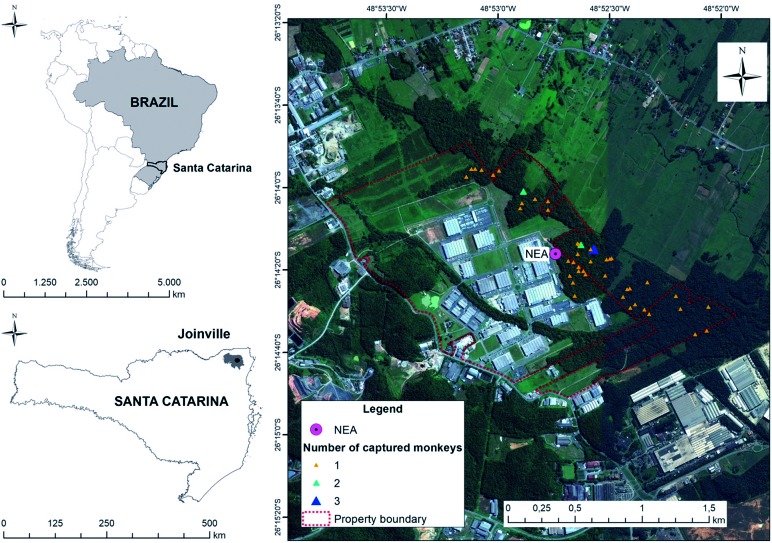
Location of the fragment of ombrophilous dense lowland forest and local where free-living Southern brown howler monkey (*Alouatta guariba clamitans*) were captured, in the city of Joinville, Santa Catarina State, South of Brazil. NEA = laboratory. Triangles represent locals where one (yellow), two (green) or three (blue) monkeys were captured. Area delimited by dashed red line corresponds to the property of the Perini Business Park where the study was developed. Image from Google Earth, Maxar technologies.

The hydrographic basin relief is constituted by alluvial-sedimentary plains with great pedogenic development, and the region is formed by a single littoral plain. The area is characterised by strongly degraded Atlantic Forest vegetation cover, which also affects watercourses. Although fragmented, the area presents secondary vegetation at an advanced stage of regeneration, where predominate zoochoric species.

According to the Köppen’s classification, the predominant climate in the region is mesothermal, humid, without dry season. The annual average relative humidity of the air is 76%. It presents a humid mesothermal subtropical climate type with hot summer. The region is greatly influenced by the relief, particularly the sea ridge, which acts as a natural barrier to the humidity brought from the ocean. For this reason, the moisture and rainfall indexes are higher than in the plateau areas.

*Procedure of capture and biological material sampling* - Two campaigns were performed to capture howler monkeys, from July to December in 2015 and 2017. The search for the animals was performed through linear transects, formed by perpendicular trails of 300 to 1,000 metres in length, with a distance of 100 metres between them, previously open for the estimation of the density of individuals in the area. We have also done an active search in the forest sites where the permanence of the animals was already known. To capture the howler monkeys, we employed an carbon dioxide (CO_2_) injector dart projector, model 70 (Dist Inject®, Brochers S.A., Gernika, Spain), using butane gas for pressure.^9^ The darts (Mini-ject®, Miltex Cirurgical instruments, Plainsboro, USA) used had a caliber of 11 mm, with wool stabiliser, 25 mm needle with bush, and a counterweight. The anesthetic used for the chemical containment was the combination of tiletamine hydrochloride and zolazepam hydrochloride at a dose of 5.5 mg/kg. The site selected for dart insertion in the animal was the lower forth, giving preference for the gluteus and thigh.^9^ The captured animals were protected during the fall with a safety net or rescued by abseiling and tree climbing.

We used a protocol of clinical evaluation, collection of biological material, and morphometric measures, adapted from the one proposed by the Chico Mendes Institute of Biodiversity Conservation (ICMBio).^10^ The records and information from each animal captured were performed, such as capture date, capture identification (ID), microchip number implanted in the subcutaneous region of the intra-scapular region, sex, and age group. The age was estimated as proposed by Carpenter to categorise the animals as non-adults (juveniles) and adults (including also subadults).^11^ Furthermore, we performed the clinical evaluation of each individual, considering rectal temperature, respiratory and heart rates, palpation of the abdomen, as well as visual inspection of the eyes and nostrils, skin, hair, among others. The effects of anesthesia were monitored every ten minutes collecting information such as heartbeat, respiratory movements, rectal temperature, and O_2_ saturation. All the procedures were performed in the place of the capture.

The morphometric parameters of the howler monkeys were measured with a pachymeter (head width, face, ear width, ear length, forearm length, arm, hand, scrotum or vulva width, scrotum or vulva length) and with a measuring tape (head-tail length, tail, chest circumference, arm circumference, thigh circumference), according to procedures standardized at CEPESBI.

Blood collection (4 mL/kg) was performed, after weighing the animal, by puncturing the femoral or brachial vein using a needle, and vacuum tubes without and with anticoagulant (5% EDTA). After collection, the tubes with blood and anticoagulant were stored refrigerated (10ºC) until the haematological examinations be performed (maximum 6 h after the collection). The blood tubes without anticoagulant were incubated in a water bath (37ºC for blood clot retraction and serum obtaining, after centrifugation at 3,000 *x g* for 5 min), for further biochemical analysis in the laboratory. All unused biological material was deposited in the collection of biological material of howler monkeys of the Centro de Pesquisas Biológicas de Indaial/SC under registration in Conselho de Gestão do Patrimônio Genético (CGEN), Process nº 02000.003226/2006-91. After the procedure, the animals were kept in the transport box for approximately 5 h after the term of the procedures and released at the same capture site after the anesthetic effect ended.

*Haematological analysis* - To perform the blood count, we used total blood in tubes containing EDTA and the analysis was done in the automated veterinary haematological counter pocH-100iV Diff® (Sysmex, Japan), immediately after collection. We performed differential count of leukocytes (neutrophils, eosinophils, basophils, lymphocytes, monocytes, and rods) by reading the blood smear slides stained with quick panoptic under an optical microscope. The quick panoptic was performed according to the manufacturer’s specifications.

*Biochemical analysis* - The biochemical exams were performed in duplicate of aliquots of serum stored in a freezer at -18ºC for up to 30 days. Dosages of the samples collected in 2015 were performed with the use of commercial kits and read in BIO-2000® photocolorimeter (Bioplus, Brazil) in FURB biochemistry laboratory. The biochemical dosages included glucose, cholesterol, triglycerides, total proteins, albumin, globulins, urea, creatinine, ALT, AST, gamma-glutamyl transferase, alkaline phosphatase, creatine kinase, and lactate dehydrogenase. Biochemical parameters were not analysed in the samples collected in 2017 because of the equipment was not available.

*Plasmodium spp. diagnosis* - We added 4 mL of RNAlater (QIAGEN, Minneapolis, USA) to 1 mL of whole blood, and 300 µL were used for DNA extraction using the QIAamp DNA mini Kit (QIAGEN, Minneapolis, USA) according to the manufacturer’s specifications. For DNA extraction control, the samples were submitted to polymerase chain reaction (PCR) for amplification of the mammalian cytochrome B gene,^12^ which amplifies a 350 bp fragment in Neotropical primates (data not shown).

The molecular diagnosis was performed by *Nested*-PCR targeting the 18S small subunit (SSU) rRNA or the mitochondrial *coxI* gene. The *Nested*-PCR reactions targeting the 18S SSU used the protocol and primers described by Snounou et al. for diagnosis of *Plasmodium* species infecting humans.^13^ The primers described for *P. malariae* were employed to identify *P. brasilianum* infection in NWPs and the primers for *P. vivax* were used to identify *P. simium* infection in NWPs, because those primers do not discriminate among these two pairs of *Plasmodium* species. Briefly, the reactions were performed in a 20 µL volume using: 100-200 ng of DNA, 0.25 μM of each primer, 10 μL of Master Mix (PROMEGA- 0.3U Taq DNA Polymerase, 200 μM each of dNTPs and 1.5 mM MgCl_2_). Amplifications were performed on the PTC-100 automatic thermocycler Version 7.0 (MJ Research Inc, Watertown, MA, USA) and the cycling conditions were, for the first reaction: a cycle of 95ºC for 5 min, 24 cycles of 58ºC for 2 min, 72ºC for 2 min, and 94ºC for 1 min, followed by a final cycling of 58ºC for 2 min, and 72ºC for 5 min, and 4ºC for unlimited time. The second reaction was performed under the same conditions, but with 29 cycles, using 1.0 μL of the amplified product of the first reaction as template DNA.

The differential diagnosis of *P. simium* in relation to *P. vivax* was based on the *Nested*-PCR of a *coxI* gene fragment and subsequent enzymatic digestion, using primers and protocol described by Alvarenga et al.^14^ Briefly, the first reaction was performed in 20 μL volume containing 0.5 μM of each primer, 2 μL of DNA, 0.2 μL of Taq DNA polymerase (5 U/μL) (Invitrogen), 0.2 mM dNTPs, and 1.5 mM MgCl_2_. PCR assays were performed in a thermocycler (Veriti 96 wells, Applied Biosystems) with the following cycling parameters: initial denaturation at 94ºC for 2 min, followed by 40 cycles of denaturation at 94ºC for 30 s, annealing at 54ºC for 20 s, extension at 72ºC for 30 s, followed by a final extension incubation at 72ºC for 2 min. The temperature was then reduced to 4ºC until the samples removal. For the second PCR, 1-3 μL of the primary product was used as template. The cycling parameters for the second PCR cycle were the same as for the first cycle. Amplified fragments were visualised by electrophoresis on 2% agarose gels in 1x TAE buffer (40 mM Tris-acetate, 1 mM EDTA) with 5 μg/mL ethidium bromide (Invitrogen) in a horizontal system (Bio-Rad) at 100V for 30 min. The differential diagnosis between *P. brasilianum* and *P. malariae* has not been available yet.

The PCR products (verified by agarose gel electrophoresis) were digested with the restriction enzyme *Hpy*CH4III (New England Biolabs, Ipswich, MA, USA). Digestion was performed in 10 μL containing 0.5 μL of the enzyme (5 U/μL), 1 μL of the enzyme buffer, and 5-7 μL of the PCR product according to its intensity in the agarose gel. The digestion was incubated at 37ºC for 3 h. The whole products of the digestion and the equivalent amount of undigested DNA were visualised in 3% agarose gel electrophoresis and examined under a UV transilluminator.

*Statistical analysis* - Analysis were performed to compare the parameter values among sex, age groups (adults and non-adults) and infected and non-infected individuals. The distribution of the parameters was measured by the Kolmogorov Smirnov and Shapiro Wilk tests. The variables with normal distribution [weight, erythrocytes, total leukocytes, neutrophils, rods, haemoglobin, hematocrit, platelets, mean corpuscular haemoglobin concentration (MCHC), cholesterol, triglycerides, total proteins, albumin, urea, ALT, Gamma glutamyl transferase, alkaline phosphatase, creatinine kinase, head-tail length, tail, face, arm circumference, thigh circumference, scrotum or vulva width, scrotum or vulva length] were analysed using the Student’s *t*-test. The variables without normal distribution [eosinophils, lymphocytes, monocytes, mean corpuscle volume (MCV), mean corpuscular haemoglobin (MCH), glucose, creatinine, AST, lactate dehydrogenase, and albumin/globulin ratio, head width, ear width, ear length, forearm length, arm length, hand, thigh, leg of the heel to the knee, foot, circumference of the thorax] were analysed using the *Mann-Whitney U* test. For the categorical variables, *Chi-square* test or *Fisher’s* exact test was used. For parameters that juvenile howler monkeys previously showed different values (JCSJ personal communication), they were excluded, those analyses were specified in the results section. The statistical difference was considered ≤ 0.05. The data were analysed in the program SPSS version 23.

*Ethical and legal considerations* - The study was approved by the Ethical Committee on the Use of Animals of the Regional University of Blumenau - FURB, under the protocol nº 012/15 and authorised by the System of Authorisation and Information in Biodiversity - SISBIO nº 43375-6.

## RESULTS

The first campaign for the capture of howler monkeys occurred from July to December 2015, totaling 22 days in the field, and 30 captured animals (29 different individuals). The second campaign was carried out in the same period of 2017, totaling 22 days in the field, and 19 captured animals (11 different individuals). The capture index in both campaigns was 1.1 monkey/day (49 monkeys/44 days).

We captured animals of both sexes and from different age groups. In total, 40 individuals (21 females and 19 males) were captured (Table I). Seven individuals were captured twice, and one was captured three times; 32 individuals were captured only once (Table II). The total number of captured animals corresponded to 30% of the total population estimated for this fragment area.

The diagnosis of *Plasmodium* spp. resulted from the combination of two molecular methodologies; initially to identify infection by *P. simium/P. vivax* and by *P. brasilianum/P. malariae*, and, subsequently, for differential diagnosis of *P. simium* infection [Supplementary data (Table I, Fig. 2)]. The positivity rate of *Plasmodium* spp. infection in the captured animals was estimated at 70% (28/40) (Table I). In 2015, the positivity was 75.86% (22/29) and in 2017 it was lower, estimated in 55.6% (10/18), considering only different individuals in each capture [Supplementary data (Table I)].

**TABLE I:** Infection by *Plasmodium* spp. in free-living Southern brown howler monkeys (*Alouatta guariba clamitans*) from Joinville, Santa Catarina State, south of Brazil

Molecular diagnosis of *Plasmodium* infection^*a*^	Sex				Age group^*c*^				Total	
	Female		Male		Adults		Non-adults				
	n	%	n	%	n	%	n	%	n	%
*P. simium*	5	23.81	4	21.05	8	23.53	1	16.67	9	22.50
*P. brasilianum/P. malariae*	1	4.76	2	10.53	1	2.94	2	33.33	3	7.50
Mixed^*b*^	8	38.10	8	42.11	14	41.18	2	33.33	16	40.00
Negative	7	33.33	5	26.32	11	32.35	1	16.67	12	30.00
Total of positives	14	66.67	14	73.68	23	67.65	5	83.33	28	70.00
Total	21		19		34		6		40	

*a*: results of combined diagnosis by *Nested*-polymerase chain reaction (PCR) for ribosomal and mitochondrial targets; *b*: infection by *P. simium* and *P. brasilianum/P. malariae*; *c*: age according to Carpenter et al.^11^

**TABLE II:** Infection by *Plasmodium* spp. in re-captured free-living Southern brown howler monkeys (*Alouatta guariba clamitans*) in Joinville, Santa Catarina State, south of Brazil

Group^*a*^	Monkey	Sex^*b*^	Age^*c*^	Sample	Date^*d*^	Infection^*e*^
(i)	1	F	A	1	07/07/2015	Mixed
				7	15/08/2015	Mixed
(i)	7	F	A	8	15/08/2015	Neg
				47	07/12/2017	Neg
(i)	9	M	A	10	17/08/2015	Pb/Pm
				38	05/08/2017	Pb/Pm
(ii)	10	F	S	11	18/09/2015	Ps
				40	01/09/2017	Neg
(ii)	17	M	A	18	17/10/2015	Neg
				34	12/07/2017	Ps
(iii)	12	M	A	13	19/09/2015	Pb/Pm
				48	08/12/2017	Ps
(iii)	20	M	J	21	27/11/2015	Pb/Pm
				44	11/11/2017	Ps
(iii)	8	M	A	9	16/08/2015	Ps
				39	06/08/2017	Mixed
				42	06/10/2017	Ps

*a*: group: (i) The diagnosis was identical in all samples; (ii) Diagnosis was negative at one time and positive at another; and (iii) The infection was diagnosed with different species at different times of blood collection. *b*: F = female; M = male; *c*: age estimated according to Carpenter, 1965: A = adult; S = subadult; J = juvenile;^(11)^ *d*: sample collection date; *e*: mixed = mixed infection (*P. simium* + *P. brasilianum/P. malariae)*; Pb/Pm = *P. brasilianum/P. malariae*; Ps = *P. simium*; Neg = negative.

There was no difference in the positivity rate of *Plasmodium* spp. infection between males and females (*Chi-square* test, p = 1.00), even when considered only adults (*Fisher’s* exact test, p = 1.00). The positivity rate of *Plasmodium* spp. infection in juveniles was 83.33% (5/6); only one animal was not infected. The positivity of infection in adults was 67.65% (23/34) (Table I). However, there was no statistical difference in the percentage of infection between the age groups (*Fischer’s* exact test, p = 0.326).

For animals captured more than once, it was observed that animals 1, 7 and 9 had the same diagnosis in both captures, however the other monkeys showed distinct diagnosis at different times [Table II, Supplementary data (Table I)]. The animal 8, which was caught three times, presented positive diagnosis for *P. simium* in 2015, in the first capture of 2017 presented mixed infection, and in its last capture (two months later) it was positive for *P. simium* again. Animal 10 was positive for *P. simium* in 2015 and was negative for *Plasmodium* infection in 2017. Animals 12 and 20 were diagnosed with *P. brasilianum/P. malariae* in 2015, and in 2017 were positives for *P. simium*. Animal 17 was diagnosed as negative in 2015, and in 2017 was positive for *P. simium*.

Females and males presented body weight of 3.91 kg (± 0.90) and 5.23 kg (± 0.73), respectively, considering only adults and subadults, excluding juveniles, because they showed weight significantly lower (*Student’s t*-test, p = 0.003). The mean values of the clinical parameters evaluated were: rectal temperature 37.37ºC (± 1.65); heart rate 154.96 bpm (± 27.40) and respiratory rate 21.41 breaths per minute (± 7.23). Upon palpation, two individuals, one infected and the other uninfected (monkey six and monkey 20 in 2017), showed splenomegaly. All monkeys had the mucosa normally colored and are well-hydrated. None statistical differences were detected in the weight, clinical parameters, and morphometric measures [Supplementary data (Table II)], when comparing infected and uninfected groups.

In the absence of reference values for free-living *Alouatta g. clamitans*, haematological (Table III) and biochemical (Table IV) parameters were analysed comparing infected and uninfected animals, considering only adults and subadults (n = 34). The juveniles (n = 6) were excluded because they showed some values significantly lower (haemoglobin - p <0.001; hematocrit - p = 0.015; monocytes - p = 0.045; urea - p = 0.015). Comparison of the parameters between monkeys infected with *P. simium* and with *P. brasilianum/P. malariae* was not possible because excluding the juveniles only one monkey was infected with *P. brasilianum/P. malariae*. The infected howler monkeys presented higher mean value of lymphocyte (p = 0.010), and ALT (p = 0.003). Analysing the animals sorted by sex, females infected with *Plasmodium* spp. presented higher mean of lymphocyte (p = 0.014), urea (p = 0.029), and ALT (p = 0.003) levels comparing to uninfected females. Infected males showed lower mean of albumin level (p = 0.049), compared to uninfected males. There was no difference in any of the haematological values of howler monkeys among those who were infected by only one *Plasmodium* species (*P. simium* or *P. brasilianum/P. malariae*) (n = 10) and those with mixed infection (n = 12). However, individuals with mixed infection had lower values of total proteins (p = 0.000) and higher levels of ALT (p = 0.009) than those infected with only one species of the parasite.

**TABLE III:** Haematological parameters of the free-living Southern brown howler monkeys (*Alouatta guariba clamitans*), infected and uninfected by *Plasmodium* spp. in Joinville, Santa Catarina State, south of Brazil

Haematological parameters	Infected (n = 22)		Uninfected (n = 12)		p^*a*^
	Mean	SD	Mean	SD		
Erythrocytes (x10^6^/µL)	4.74	0.67	4.56	0.65	0.464
Haemoglobin (g/µL)	9.93	1.51	9.92	1.23	0.975
Hematocrit (%)	33.35	4.22	32.96	3.86	0.787
Mean Corpuscular Hemoglobin Concentration (g/µL)	29.70	1.77	30.11	1.78	0.533
Mean Corpuscular Volume (fL)	70.59	4.16	72.72	6.36	0.312
Mean Corpuscular Hemoglobin (pg)	20.98	1.90	21.97	3.13	0.331
Platelets (x10³/µL)	57.86	34.39	76.33	50.53	0.274
Leucocytes (x10³/µL)	6.43	2.66	6.02	2.36	0.647
Neutrophils (x10³/µL)	3.23	0.46	3.44	0.40	0.270
Band Cells (x10³/µL)	0.19	0.06	0.23	0.06	0.320
Eosinophils (x10³/µL)	0.14	0.04	0.16	0.05	0.557
Lymphocytes (x10³/µL)^b^	2.80	0.48	1.76	0.29	0.010
Monocytes (x10³/µL)	0.12	0.03	0.10	0.03	0.423

*a*: *Student’s t*-test and *Mann-Whitney U* test, comparing infected and uninfected animals (p ≤ 0.05 in bold); *b*: significantly higher in infected females compared to uninfected females (p = 0.014). In this analysis, we only included adults and subadults. SD = standard deviation.

**TABLE IV:** Biochemical parameters of samples collected in 2015 from free-living Southern brown howler monkeys (*Alouatta guariba clamitans*), infected and uninfected by *Plasmodium* spp. in Joinville, Santa Catarina State, south of Brazil

Biochemical Parameters	Infected (n = 17)		Uninfected (n = 7)		p^*a*^
	Mean	SD	Mean	SD	
Alanine amino tranferase (UI/L)^*b*^	30.97	10.07	16.93	6.55	0.003
Albumin (g/dL)^*c*^	3.28	0.68	3.59	0.50	0.298
Albumin/globulin ratio	0.95	0.23	0.93	0.31	0.745
Alkaline phosphatase (UI/L)	163.06	59.26	134.71	34.35	0.252
Aspartate amino tranferase (UI/L)	103.88	39.36	107.35	66.82	0.418
Cholesterol (mg/dL)	78.65	26.63	88.57	31.41	0.439
Creatine kinase (UI/L)	589.29	378.16	653.00	150.48	0.561
Creatinine (mg/dL)	1.08	0.47	0.84	0.29	0.494
Gamma-glutamyl transferase (UI/L)	54.41	15.91	54.00	22.57	0.960
Globulins (g/dL)	3.62	0.83	4.11	1.00	0.221
Glucose (mg/dL)	97.24	35.91	91.14	33.22	0.619
Lactate dehydrogenase (UI/L)	644.21	424.95	491.07	286.71	0.318
Total proteins (g/dL)^*d*^	6.98	1.27	7.54	0.74	0.192
Triglycerides (mg/dL)	126.65	56.56	124.50	89.81	0.944
Urea (mg/dL)^*e*^	34.47	23.98	27.43	16.82	0.488

*a*: *Student’s t*-test and *Mann-Whitney U* test, comparing infected and uninfected animals, considering assumption of equal variances (p ≤ 0.05 in bold); *b: s*ignificantly higher in infected females compared to uninfected females (p = 0.003), and higher also in animals with mixed infection (*P. simium* and *P. brasilianum/P. malariae*) compared to those infected with only one species of *Plasmodium* (p = 0.009); *c*: significantly lower in infected males than in uninfected males (p = 0.044); *d*: significantly lower in animals with mixed infection (*P. simium* and *P. brasilianum/P. malariae*) compared to those infected with only one species of *Plasmodium* (p = 0.000); *e*: significantly higher in infected females compared to uninfected females (p = 0.029). In this analysis, only adults and subadults caught in 2015 were included, because of differences in some parameters for juveniles (see text for more details), samples from 2017 were not included because the equipment for biochemical analysis was not available that time. SD: standard deviation.

## DISCUSSION

*Plasmodium brasilianum* has been described as able to infect NWPs in the Amazon and Atlantic Forest biomes, in Brazil.^3^ ^,^ ^6^ ^,^ ^7^ ^,^ ^8^ This species infects naturally a wide range of Neotropical primates, including species from all families of NWPs.^3^ ^,^ ^15^ This parasite is widely diffused from Central America to the South of Brazil.^4^ ^,^ ^15^ ^,^ ^16^ ^,^ ^17^ ^,^ ^18^

*Plasmodium simium* is restricted to the Atlantic Forest in Southern and Southeastern Brazil, where it has been reported in the states of Espírito Santo, Rio de Janeiro, São Paulo, Santa Catarina and Rio Grande do Sul.^3^ ^,^ ^7^ ^,^ ^8^ ^,^ ^18^ ^,^ ^19^ Thus far, only few species of NWPs were diagnosed with natural *P. simium* infection.^3^ ^,^ ^17^ ^,^ ^19^

The percentage of *Plasmodium* spp. infection observed in the present study was the greatest one found in the literature for species of NWP. The highest value was described for Guaiba municipality in Rio Grande do Sul State (57.1%).^18^ In Santa Catarina State the rates of *Plasmodium* spp. infection previously described ranged from 35% to 46%.^3^ ^,^ ^8^ ^,^ ^17^ However, it should be considered that the methodologies used in these studies have different sensitivities. In accordance with other studies in the Atlantic Forest,^7^ ^,^ ^8^ we identified that the positivity of *P. simium* infection (22.5%) was higher than that of *P. brasilianum* (7.5%).

The high positivity rate found in Joinville can be attributed to the forest ecosystem of the region. The dense ombrophilous lowland forest is characterised by the presence of large numbers of bromeliads, plants that accumulate water and function as natural breeding grounds for anopheline species of the subgenus *Kerteszia*, which are responsible for the parasite transmission.^20^

In the howler monkeys analysed in this study, that were sampled at different times, we observed three different patterns: (i) the results of diagnosis were the same in all samples from the same animals (animals 1, 7, and 9); (ii) infection was negative at one time and positive at another (animals 10 and 17); and (iii) the infection was diagnosed with different species in different captures (animals 8, 12 and 20). In the first group, animal 1 was positive twice in an interval of five weeks and animal 9 with an interval of two years, which may suggest a chronic infection, as proposed by Deane^17^ and Erkenswick et al.^16^ However, in order to confirm this hypothesis, it would be necessary to showed that the parasites in the same host are genetically identical at the different times. In the second group, animal 17 became infected between 2015 and 2017, and animal 10 became negative in this period, which may be due to self-cure, already described for *P. simium*.^17^ In the third group, the diagnosis varied among samples from the same individual at different times, which might be due to transitory infection with one *Plasmodium* species that are self-cured and posterior new infection with another *Plasmodium* species*.* Another possible explanation for this result could be the sensitivity of the diagnosis methods that sometimes does not detect very low parasitaemia, mainly in mixed infections.^13^ ^,^ ^14^

Our results showed an increase in lymphocytes in the blood of howler monkeys infected with *Plasmodium* spp*.* Lymphocyte augmentation was already reported in splenectomised common marmosets (*Callithrix jacchus*) infected in laboratory with *P. brasilianum*.^21^ In general, human patients infected with *P. vivax* tend to present a significantly lower lymphocyte counts.^22^ However, our results agreed with other authors, such as Lacerda et al.,^23^ in which the mean lymphocyte count was elevated in 29.6% of *P. vivax* patients from Brazilian endemic area.

Although the number of samples included in biochemical analysis was not high, we obtained some very interesting results different statistically comparing infected and non-infected monkeys. Moreover, the difficulty of free-living monkeys capture and the scarcity of data from those animals justify the importance of the obtained results. Among the infected males, low levels of albumin was observed, which also corroborates the report made by Costa et al.,^8^ where howler monkeys with symptoms suggestive of malaria presented hypoalbuminemia. The hypoalbuminemia may be a consequence of albumin uptake and degradation by the parasites during intraerythrocytic growth, and differentiation.^24^ It could also be due to a decrease in the synthesis of albumin by hepatocyte degradation in the presence of parasites. Increased serum urea, as we observed for infected female monkeys, has been reported in human patients infected with *P. falciparum*, and its values here higher in infected women compared to infected men.^25^ High levels of urea were also associated to thrombocytopenia in *P. vivax* infected patients.^26^

The augmented levels of the hepatic enzyme ALT in infected howler monkeys corroborate the unique report of an animal of the same species (*A. guariba clamitans*) with symptoms suggestive of malaria.^8^ Abnormalities in liver function have been reported in patients infected with different species of *Plasmodium*, generally without significant clinical complications.^22^ In the present work, infected animals showed higher levels of ALT in analyses including all animals, in the analysis comparing infected and uninfected females, and also in mixed infections compared to single one. The low values of the total proteins in animals with mixed infection, when compared to the animals with only one *Plasmodium* species infection*,* reinforce the suggestion that the infection might cause insufficiency in the synthesis of proteins due to possible liver damage. Furthermore, it suggested that the howler monkeys with mixed infection are under a greater challenge to maintain homeostasis because of the additive effect of the presence of the two species of parasites.^27^ Altogether, these data suggest that *Plasmodium* spp. infection may results in hepatic disturbance in NWPs despite of none clinical alteration due to infection was observed. The splenomegaly upon abdominal palpation detected in two animals was not related to *Plasmodium* infection, because only one of them was infected by *Plasmodium*.

It is not yet known whether under natural conditions NWPs infected by *Plasmodium* spp. may develop malaria. Most of the studies that characterise the clinical features of malaria in monkeys were generally performed after experimental infection in splenectomised animals.^2^ Natural infection by *P. simium* is characterised by low parasitaemia; however, after splenectomy parasitaemia increases.^17^ In those animals was observed weakness, fever, anemia, jaundice, and renal failure.^17^ Most of the time, the infection is self-controlled after a few weeks, but in some cases the animals evolved to death due to the increase of the parasitaemia. The symptoms observed in naturally infected howler monkeys were similar to those resulting from experimental infections.^17^ The infection by *P. simium* in neotropical primates is sub patent, chronic, and usually asymptomatic, probably due to an action of the immune system controlling the parasitaemia, and consequently avoiding the changes resulting from the infection. One of the few descriptions of clinical signs and haematological changes suggestive of *Plasmodium* natural infection in NWPs was performed by Costa et al.^8^ Therefore, naturally infected howler monkeys may show clinical signs of malaria, particularly under stress conditions.^8^

The influence of *Plasmodium* spp. infection for the health of NWP host is not fully known yet. This study allow the conclusion that, although the howler monkeys were generally asymptomatic, alterations in the haematological and biochemical parameters were observed in infected monkeys, which may have an effect on the health of the animals, particularly under stress conditions.^8^ Since stress could cause immunosuppression and increase the impact of diseases, the conservation of the endangered *A. guariba clamitans* is highly dependent of the maintenance of its habitat and wildlife corridors to avoid unnecessary stress.^28^ ^,^ ^29^ It is also necessary to evaluate *Plasmodium* spp*.* infection among the risks during its translocation, including the molecular analysis, as presented here, in the Normative Instruction number 23 of the Brazilian Institute of Environment and Natural Renewable Resources (http://www.ibama.gov.br/component/legislacao/?view=legislacao&legislacao=134768). Moreover, considering the large number of other infections that free-living monkey could have the influence of co-infections with *Plasmodium* spp. infections still need further studies.

The high positivity rates of *Plasmodium* spp. infection in NWPs, as well as the characteristics of the vegetal cover of the study area, favorable for the proliferation of vectors, constitute a worrying eco-epidemiological scenario of malaria in the region. The growth of contact between the human host and the forest areas, mainly through ecotourism,^30^ increases the potential for autochthonous cases of human malaria in the municipality. The mild symptoms of human malaria in the Atlantic Forest and available treatment in the public health system of Brazil (same used for *P. vivax*) suggest that the main requirements for malaria prevention and control in those areas are environmental education strategies for local population and tourists, and the epidemiological surveillance of malaria. Therefore, the inclusion of Atlantic Forest as area of malaria transmission will allow that physicians request malaria diagnosis in cases of fever and, if needed, the treatment, must be promptly implemented.
