# Supplementary material for: Plasmodium infection and its association with biochemical and haematological parameters in free-living Alouatta guariba clamitans (Cabrera, 1940) (Primates: Atelidae) in Southern Brazil
Source: Mem Inst Oswaldo Cruz. 2020 Jan 31;114:e190210. doi: 10.1590/0074-02760190210 (PMC6996493; doi:10.1590/0074-02760190210)
Supplement: Supplementary file 1 [file 1678-8060-mioc-114-e190210-s.pdf]

TABLE I

Diagnosis of infection by *Plasmodium* spp. of the free-living Southern brown howler monkeys (*Alouatta guariba clamitans*) in Joinville, Santa Catarina State, south of Brazil, by the year of capture

Year	Animal number	Sample	Diagnosis 1[a]	Diagnosis 2[b]	Final combined result[c]
2015	1	1	Pm	Ps, NPs	mixed
	2	2	Pv, Pm	Ps	mixed
	3	3	Pv, Pm	Ps, NPs	mixed
	4	4	Pm	Ps, NPs	mixed
	5	5	Pv, Pm	Ps	mixed
	6	6	Neg	Neg	Neg
	1	7	Pv, Pm	Ps	mixed
	7	8	Neg	Neg	Neg
	8	9	Pv	Ps	Ps
	9	10	Pm	Neg	Pb/Pm
	10	11	Pv	Ps	Ps
	11	12	Neg	Neg	Neg
	12	13	Pm	Neg	Pb/Pm
	13	14	Pv, Pm	Ps	mixed
	14	15	Pv	Ps	Ps
	15	16	Neg	Neg	Neg
	16	17	Pv, Pm	NA	mixed
	17	18	Neg	Neg	Neg
	18	19	Neg	Neg	Neg
	19	20	Pv	Ps	Ps
	20	21	Pm	NA	Pb/Pm
	21	22	Pv	Ps	Ps
	22	23	Neg	Neg	Neg
	23	24	Pm	NA	Pb/Pm
	24	25	Pv	Ps	Ps
	25	26	Pv, Pm	Ps	mixed
	26	27	Pv, Pm	Ps	mixed
	27	28	Pv, Pm	Neg	mixed
	28	29	Pv, Pm	Ps	mixed
	29	30	Pm	NPs	Pb/Pm
2017	30	31	Neg	Ps	Ps
	31	32	Neg	Ps	Ps
	32	33	Neg	Ps	Ps
	17	34	Pv	Ps	Ps
	33	35	Neg	Neg	Neg
	34	36	Neg	Neg	Neg
	35	37	Neg	Neg	Neg
	9	38	Neg	NPs	Pb/Pm
	8	39	Neg	Ps, NPs	mixed
	10	40	Neg	NA	Neg
	36	41	Neg	NA	Neg
	8	42	Pv	Ps	Ps
	37	43	Pv, Pm	Ps	mixed
	20	44	Neg	Ps	Ps
	38	45	Neg	Neg	Neg
	39	46	Neg	Neg	Neg
	7	47	Neg	NA	Neg
	12	48	Pv	Ps	Ps
	40	49	Pm	Ps, NPs	mixed

a: diagnosis by *Nested*-polymerase chain reaction (PCR) based on the 18S small subunit (SSU) rRNA (Snounou et al.),[13] positive results using primers for *Plasmodium vivax* (Pv) or *P. malariae* (Pm) or negative results using all primers (Neg); *b*: differential diagnostics between *P. simium* (Ps) and other *Plasmodium* species (non-*P. simiun* - NPs) based on the amplification by *Nested*-PCR of the *coxI* gene and digestion with *Hpy*-CH4III (Alvarenga et al.);[14] *c*: the final result was based on the combination of the results from both diagnostic methodologies and in all samples of the same animals. Animals 8, 12 and 20 were considered as presenting mixed infections and animals 10 e 17 were considered infected by *P. simium*. Mixed: *Plasmodium simium* + *P. brasilianum/P. malariae*; Pb/Pm: *Plasmodium brasilianum/P. malariae*; Ps: *Plasmodium simium*; Neg: negative; NA: Not amplified by PCR.

CC BY

TABLE II

Morphometric parameters of free-living Southern brown howler monkeys (*Alouatta guariba clamitans*), in Joinville, Santa Catarina State, South of Brazil, according to sex and infection by *Plasmodium* spp.

	Female					Male				
	Infected (N = 11)		Uninfected (N = 7)			Infected (N = 11)		Uninfected (N = 5)		
Morphometric parameter	Mean	SD	Mean	SD	p[a]	Mean	SD	Mean	SD	p[a]
1 - Head-tail length	46.00	3.61	47.43	3.64	0.430	52.91	4.11	52.80	2.59	0.950
2 - Tail	51.00	2.37	52.57	4.79	0.364	55.36	4.18	54.70	1.98	0.673
3 - Head width	5.74	0.34	5.97	0.43	0.263	6.55	0.50	6.66	0.31	0.587
4 - Face	5.73	0.42	5.57	0.28	0.350	6.73	0.51	6.82	0.51	0.745
10a - Ear width	2.29	0.51	2.14	0.48	0.375	2.45	0.58	2.22	0.16	1.000
10b - Ear length	2.94	0.39	2.94	0.39	0.973	2.85	0.60	3.20	0.07	0.441
12 - Forearm length	14.15	0.81	14.86	0.90	0.115	16.00	0.89	16.30	0.84	0.583
13 - Arm	14.54	0.78	14.57	1.40	0.791	16.14	0.84	16.70	1.30	0.412
15 - Arm circumference	11.09	1.11	11.21	0.91	0.724	13.23	1.60	14.20	2.28	0.422
14 - Hand	9.67	2.92	10.79	0.95	0.536	11.64	0.50	11.60	0.42	0.827
16 - Thigh	14.83	0.72	15.43	0.77	0.479	16.70	1.30	16.32	1.10	0.588
19 - Thigh circumference	14.36	1.21	15.14	1.07	0.179	16.77	1.84	16.80	1.30	0.827
17 - Leg from knee to heel	14.60	0.77	15.07	1.17	0.479	16.23	0.93	16.40	0.89	0.827
18 - Foot	12.86	0.50	13.07	0.79	0.659	14.09	0.49	14.20	0.57	0.743
A - Scrotum Length	2.66	0.49	2.46	0.46	0.384	3.00	0.55	2.56	0.59	0.201
B - Scrotum Width	2.26	0.47	2.21	0.27	0.781	2.38	0.48	2.70	0.75	0.421
11 - Thorax circumference	31.59	3.74	32.57	3.05	0.552	36.13	3.19	36.60	0.55	0.756

a: none were statistically significant ($p \leq 0.05$); SD: standard deviation. In this analysis were included only adults and subadults.

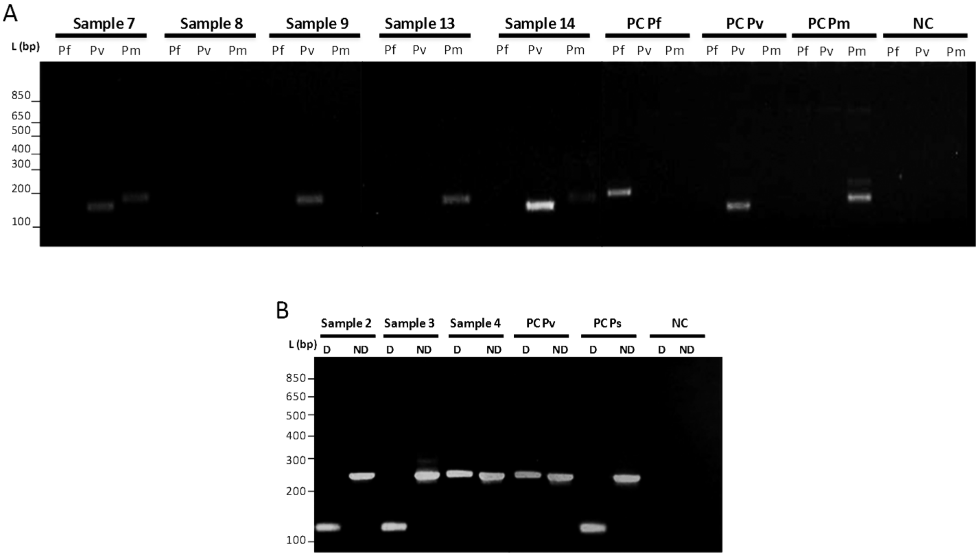

Representative results of *Plasmodium* species diagnostic of some of the non-human free-living *Alouatta guariba clamitans* captured in Joinville/SC. (A) Diagnostic by *Nested*-polymerase chain reaction (PCR) [18S small subunit (SSU) rRNA] according to Snounou et al.[13] of five samples: sample seven (animal 1), sample eight (animal 7), sample nine (animal 8), sample 13 (animal 12) and sample 14 (animal 13). (B) *Nested*-PCR-restriction fragment length polymorphism (RFLP), according to Alvarenga et al.[14] diagnostic of three samples: sample two (animal 2), sample three (animal 3) and sample four (animal 4). PC Pf: positive control for *P. falciparum*; PC Pv: positive control for *P. vivax*; PC Pm: positive control for *P. malariae*; PC Ps: positive control for *P. simium*; NC: negative control; D: after digestion with the *Hpy*CH4III enzyme; ND: non digested amplified product.
